# Supplementary material for: Providing a detailed estimate of mortality using a simulation-based collision risk model
Source: PLoS One. 2022 Nov 17;17(11):e0276757. doi: 10.1371/journal.pone.0276757 (PMC9671428; doi:10.1371/journal.pone.0276757)
Supplement: Attachment — Submitted filename: Response to Reviewers.docx [file pone.0276757.s001.docx]

Dear Editor,

Please find below our responses to the reviewer comments. We have outlined our replies to each of the points raised by the reviewers in blue and referenced the lines within the manuscript where changes have been made.

We found the review useful in enhancing the manuscript and thank the editor and reviewers for considering our paper and aiding in its improvement.

Yours faithfully,

Nicholas Horne

Responses to Reviewer's Questions

**Comments to the Author**

1. Is the manuscript technically sound, and do the data support the conclusions?

The manuscript must describe a technically sound piece of scientific research with data that supports the conclusions. Experiments must have been conducted rigorously, with appropriate controls, replication, and sample sizes. The conclusions must be drawn appropriately based on the data presented.

Reviewer #1: Yes

Reviewer #2: Partly

Reviewer 2 provides further information on their response, below. We have addressed them in detail, below..

2. Has the statistical analysis been performed appropriately and rigorously?

Reviewer #1: Yes

Reviewer #2: No

With respect to Reviewer 2, the paper does not incorporate a hypothesis driven statistical analysis, rather the analysis of data is explanatory in nature, we expect this is why reviewer 2 has responded no here. We note that Reviewer 2, below, asks that we clearly demonstrate the advances in analytical methods, which we have done. We provide more information on this below in response to their specific question. .

3. Have the authors made all data underlying the findings in their manuscript fully available?

The [PLOS Data policy](https://eur02.safelinks.protection.outlook.com/?url=http%3A%2F%2Fwww.plosone.org%2Fstatic%2Fpolicies.action%23sharing&data=05%7C01%7CN.Horne%40qub.ac.uk%7C5aa2239456ef4454e9c408da6a6acec0%7Ceaab77eab4a549e3a1e8d6dd23a1f286%7C0%7C0%7C637939305262863484%7CUnknown%7CTWFpbGZsb3d8eyJWIjoiMC4wLjAwMDAiLCJQIjoiV2luMzIiLCJBTiI6Ik1haWwiLCJXVCI6Mn0%3D%7C3000%7C%7C%7C&sdata=dcGKctH43s%2F0OKtZSTW%2B8eBEegh8qE%2FIPt%2B6SCH9en0%3D&reserved=0) requires authors to make all data underlying the findings described in their manuscript fully available without restriction, with rare exception (please refer to the Data Availability Statement in the manuscript PDF file). The data should be provided as part of the manuscript or its supporting information, or deposited to a public repository. For example, in addition to summary statistics, the data points behind means, medians and variance measures should be available. If there are restrictions on publicly sharing data—e.g. participant privacy or use of data from a third party—those must be specified.

Reviewer #1: Yes

Reviewer #2: No

The data has been uploaded to a github repository and access provided through the resubmission process.

4. Is the manuscript presented in an intelligible fashion and written in standard English?

PLOS ONE does not copyedit accepted manuscripts, so the language in submitted articles must be clear, correct, and unambiguous. Any typographical or grammatical errors should be corrected at revision, so please note any specific errors here.

Reviewer #1: Yes

Reviewer #2: Yes

5. Review Comments to the Author

Please use the space provided to explain your answers to the questions above. You may also include additional comments for the author, including concerns about dual publication, research ethics, or publication ethics. (Please upload your review as an attachment if it exceeds 20,000 characters)

**Reviewer #1**

It is an interesting study. A few comments are presented as follows:

1. The contributions of this study are suggested to add.

Descriptions on the significance of the contributions this study has made have been made clearer and expanded upon in the abstract, introduction and discussion. We have now included how this method is novel and is a step-change beyond previous iterations of this model, and how this improvement will benefit key stakeholders (e.g., TEC industry, statutory nature conservation bodes, and the regulator). Please see lines 23, 33, 35-36, 101-114, 316-321, 283-298, 367-368, 371, 383-395, 400-401.

2. How to guarantee the accuracy of simulation?

This model has been published previously with explanations on the accuracy of the simulations. Reference to this peer-reviewed work has now been included in the text of this manuscript. (Lines 127-128)

3. The writing needs more work.

The grammar and spelling have been checked carefully throughout the manuscript and any superfluous text has been removed.

**Reviewer 2 comments**

Reviewer #2: This study estimated the mortality of collision risk between a device and animal using a simulation-based collision risk model. Overall, this study is well organized and can be easily understood. However, this study did not clearly demonstrated the significant contribution in this area.

The significant contributions and novelty of this work have been expanded upon in the text throughout the manuscript and we have now included a summary section in the discussion highlighting the contribution. Please see lines 23, 33, 35-36, 101-114, 316-321, 283-298, 367-368, 371, 383-395, 400-401.

A number of studies have been conducted in this area. This paper can be further improved by more clearly demonstrating the advances in analytical methods, data sources and identification of new significant variables, over the existing studies.

We have made edits in the text to outline how the methods in this manuscript are different and an advancement on other methods (which also addresses, in part, the comment above from Reviewer 1) and an emphasis on the importance of the ability to now extract more detailed information on what we have now demonstrated are significantly important variables when predicting collision risk (i.e. speed of collision and location of the collision on the body of the animal). Please see lines 32, 287-290, 437.

What are the new and different findings compared with those from previous studies, and why are these different finding more reasonable?

As per Reviewers 1 comments, descriptions on the significance of the contributions this study have been made clearer and expanded upon in the abstract, introduction and discussion (Lines 23, 33, 35-36, 101-114, 316-321, 283-298, 367-368, 371, 383-395, 400-401). We have now included how this method is different and is a step-change beyond previous iterations of this model, and how this improvement will benefit key stakeholders (e.g., TEC industry, statutory nature conservation bodes, and the regulator). Please see lines 330-332, 383-395, 400-401.
